# No Evidence That Articulatory Rehearsal Improves Complex Span Performance

**DOI:** 10.5334/joc.103

**Published:** 2020-05-12

**Authors:** Alessandra S. Souza, Klaus Oberauer

**Affiliations:** 1 Department of Psychology, University of Zurich, CH

**Keywords:** articulatory rehearsal, complex span, training, working memory, overt rehearsal

## Abstract

It is usually assumed that articulatory rehearsal improves verbal working memory. Complex span is the most used paradigm to assess working memory functioning; yet, we still lack knowledge about how participants rehearse in this task, and whether these rehearsals are beneficial. In Experiment 1, we investigated the patterns of naturally occurring overt rehearsals in a complex span task requiring processing of a non-verbal distractor task. For comparison, another group of participants completed a matched simple span task with an unfilled delay in between the memoranda. Time permitting, participants rehearsed the memory list in forward serial order, a strategy known as cumulative rehearsal. The degree of cumulative rehearsal was correlated with recall accuracy in both span tasks. Rehearsal frequency was, however, reduced in complex span compared to simple span. To assess the causal role of rehearsal in complex span, we trained a group of participants in a cumulative rehearsal strategy in Experiment 2. This instruction substantially increased the prevalence of cumulative rehearsals compared to a control group. However, the increase in cumulative rehearsal did not translate into an increase in recall accuracy. Our results provide further evidence that rehearsal does not benefit working memory performance.

When asked to briefly hold in mind a random list of digits, letters, or words – such as a phone number or a pin-code – people often repeat this information over and over to themselves, a behavior known as *articulatory rehearsal* (Baddeley, 1986, 2012; Logie, 2011). Why do people engage in this behavior? Rehearsal is commonly thought to protect memory representations from forgetting in working memory (WM). WM is a limited-capacity system that keeps information accessible for ongoing cognitive processing. Despite the widespread belief that rehearsal is beneficial, there is limited evidence linking rehearsal causally to WM performance (for reviews see Lewandowsky & Oberauer, 2015; Oberauer, 2019). This is particularly true for complex span, which is one of the most used tasks to assess WM functioning (Conway et al., 2005). The goal of the present study is to fill this gap by assessing when and how participants rehearse in complex span, and what effect it has on recall accuracy.

## Articulatory Rehearsal in WM tasks

Span tasks are the most commonly used tasks to assess WM performance. In simple span tasks, memory items are presented sequentially for an immediate serial recall test. Complex span tasks differ from simple span only in that the memory items are interleaved with a distractor task. For verbal span tasks, articulatory rehearsal is the most common self-reported strategy, occurring in about one-third to one-half of the trials (Bailey, Dunlosky, & Kane, 2011; Chevalère, Lemaire, & Camos, 2020; Dunlosky & Kane, 2007). This finding is consistent with the assumption that rehearsal contributes similarly to recall in simple and complex span tasks (Bailey et al., 2011; Camos, Mora, & Oberauer, 2011; Hudjetz & Oberauer, 2007; Mora & Camos, 2013; Ricker & Cowan, 2010; Unsworth & Engle, 2007; Vergauwe, Camos, & Barrouillet, 2014).

Recently, Camos and colleagues argued that people bring two maintenance processes to bear in verbal WM tasks: rehearsal and refreshing (Camos & Barrouillet, 2014; Camos, Lagner, & Barrouillet, 2009; Vergauwe et al., 2014). These two processes are assumed to operate independently and in parallel, and both are said to contribute additively to memory performance. Articulatory rehearsal is assumed to involve the speech-production system, and accordingly it has been found to engage the same brain regions involved in speech production (Awh et al., 1996; Chen & Desmond, 2005; Logie, Venneri, Sala, Redpath, & Marshall, 2003; Schumacher et al., 1996). As such, it can only be used to maintain information that can be spoken. In contrast, refreshing is assumed to be a domain-general mechanism relying on attention that can be used to maintain any type of information (Camos et al., 2018). Distractor processing in complex span is assumed to disrupt refreshing to the degree that the distractor task requires central attention, but it only disrupts rehearsal if it also requires articulation. According to this view, participants can rehearse and refresh in simple span, whereas in complex span the nature of the distractor task will determine to which extent participants can use refreshing and rehearsal. Verbal distractor tasks may reduce reliance on both types of processes, but a non-verbal distractor task should reduce reliance on refreshing but not rehearsal. To the best of our knowledge, no study to date assessed how people rehearse in complex span and to which extent non-verbal distractor processing disrupts rehearsal.

## Linking rehearsal and recall in span tasks

The evidence linking rehearsal to WM performance is scarce. Studies can be organized into three categories that differ in the strength of the evidence they provide for the role of rehearsal. One class of evidence comes from studies inferring the use of rehearsal from manipulations of the demand on articulation. One such manipulation is articulatory suppression. In this procedure, participants are required to articulate aloud irrelevant information (e.g., “ba bi bu” or “the the the”) while encoding and/or maintaining the memoranda. This leads to worse performance compared to silent study. Other commonly employed manipulations are variations in word length (assumed to vary pronunciation time) or phonological similarity (assumed to increase confusion of articulated words that are phonologically similar). All these manipulations yield worse performance in WM tasks (Baddeley, Lewis, & Vallar, 1984; Larsen & Baddeley, 2003). None of these manipulations, however, show that people were engaging in rehearsal, and they all can be explained in other ways than assuming that the manipulation affects rehearsal. For example, articulatory suppression introduces interference through irrelevant information, and this interference alone can explain the performance impairment induced by it (Lewandowsky & Oberauer, 2015). Furthermore, selection of sets of words that differ along one relevant dimension (e.g., word length) does not guarantee that these words do not differ in other regards. For instance, word-length co-varies with orthographic neighborhood-size, and this variable was found to explain the effect of word-length observed in previous studies (Jalbert, Neath, Bireta, & Surprenant, 2011; Jalbert, Neath, & Surprenant, 2011).

The second class of evidence comes from the direct observation of rehearsal using overt rehearsal protocols (Rundus & Atkinson, 1970). In this protocol, participants are asked to report aloud their rehearsals on-line during presentation of the memory list. Tan and Ward (2008) recorded rehearsal patterns in a simple span task. They varied the presentation rate of the words (1, 2.5, or 5 s per word) and observed that rehearsal was more frequent with slower presentation rates. Furthermore, at slower rates the most common strategy was to repeat the words in their order of presentation, a strategy termed cumulative rehearsal (see also Bhatarah, Ward, Smith, & Hayes, 2009; Souza & Oberauer, 2018). The degree of cumulative rehearsal was correlated with recall accuracy in these studies. Souza and Oberauer (2018) replicated these findings and computed the meta-analytic correlation between cumulative rehearsal and recall across their studies (which also involved simple span) and the one reported by Tan and Ward (2008). The overall correlation was positive and of medium size (*r* = 0.46). This pattern is consistent with rehearsal being beneficial for recall in simple span tasks. Evidence from complex span tasks is, nevertheless, still lacking.

The positive correlation between serial recall and cumulative rehearsal is not sufficient to establish a causal effect of rehearsal on WM. Causality can only be inferred from the direct manipulation of the occurrence of rehearsal. Hence the third and more definitive class of evidence for a role of rehearsal is provided by studies that instructed different rehearsal patterns and assessed their effect on WM. Palmer and Ornstein (1971) observed that a cumulative rehearsal instruction improved accuracy in a probed recall test compared to rehearsals of pairs of items, or rehearsals of just the last presented item (so-called fixed rehearsal strategy). Nishiyama and Ukita (2013) showed a rehearsal benefit (of cumulative or fixed rehearsal strategies) for the maintenance of non-words for a free recall test in comparison to a condition with limited rehearsal time. Estes (1991), in contrast, observed a cost of an overt rehearsal period inserted prior to serial recall.

The only study to investigate the causal effect of rehearsal on simple span was conducted by Souza and Oberauer (2018). Across two experiments, they compared a free-rehearsal baseline against a condition in which participants were instructed to rehearse the memoranda cumulatively, or to use a fixed-rehearsal strategy. In all conditions, rehearsals were carried out aloud, recorded, and coded by the experimenter. The instructions were effective in increasing the tendency to rehearse overall, and in increasing the frequency of the instructed rehearsal patterns. Albeit the positive correlation between cumulative rehearsal and recall obtained in the free baseline, increasing the extent of cumulative rehearsal (by about one item) did not improve performance. Fixed rehearsals, in contrast, impaired recall compared to the free-rehearsal baseline. These experiments indicate that rehearsal has a causal effect on recall in simple span, but its effect is far from beneficial.

In a third experiment, Souza and Oberauer (2018) tested whether the natural occurrence of rehearsals in the free-rehearsal baseline already reflects the best performance one can achieve through rehearsal by comparing it to an articulatory suppression condition. In this new experiment, participants were presented the free-rehearsals performed by a participant in one of the previous experiments. In the suppression condition, the rehearsals were replaced by the utterance of “babibu”. Performance in the rehearsal and suppression conditions did not differ, indicating that the correlation between rehearsal and recall observed under the free rehearsal conditions does not reflect a causal effect of rehearsal on recall. Altogether, the findings of Souza and Oberauer (2018) place low plausibility on the assumption that rehearsal is beneficial to WM performance.

To the best of our knowledge, only one study manipulated rehearsal in a complex span task. In the study of Turley-Ames and Whitfield (2003), complex-span performance was assessed before and after a cumulative rehearsal instruction was provided. Participants were instructed to process the distractors quickly and devote the remaining processing time for rehearsal. This instruction improved recall compared to a control group. However, this effect could be explained by the larger amount of time participants devoted to processing of the memoranda compared to the distractors. In a second experiment, time for processing the memoranda was controlled in both the rehearsal and control groups, and the benefit of the rehearsal instruction vanished.

## The present study

The main goals of the present study were two-fold. First, in Experiment 1 we measured when and how participants rehearse in a complex span task involving the processing of a non-verbal distractor task, and how rehearsal changes as function of the time available for rehearsal. Second, in Experiment 2 we assessed the causal role of rehearsal by training participants in the use of a cumulative rehearsal strategy.

To foreshadow our results, processing of a non-verbal distractor task reduced the frequency of cumulative rehearsals spontaneously carried out in complex span, yet the length of cumulative rehearsal was positively correlated with recall accuracy, replicating the positive association observed for simple span (Souza & Oberauer, 2018; Tan & Ward, 2008). Instructing cumulative rehearsal in Experiment 2 increased the prevalence of this strategy. Nevertheless, recall performance did not improve. These results further challenge the view that rehearsal is beneficial for WM performance.

## Experiment 1

The main goal of the present experiment was to directly observe the frequency and patterns of spontaneous rehearsals carried out in a complex span task having words as memoranda and a spatial task as the distractor activity. Whereas participants in complex-span studies report rehearsal as a common strategy (Bailey et al., 2011; Dunlosky & Kane, 2007), there is no study monitoring rehearsal online during complex span. Experiment 1 served to gauge how often which patterns of rehearsal are carried out in this task. We also aimed to certify that a cumulative rehearsal strategy is positively correlated with recall (replicating previous studies; Souza & Oberauer, 2018; Tan & Ward, 2008), making it a valid candidate for our manipulation of rehearsal in Experiment 2.

To directly measure rehearsal, we asked participants to carry out their rehearsals aloud (Overt Rehearsal groups). There has been a concern regarding how similar overt rehearsal is to covert rehearsal. For example, Cowan and Vergauwe (2015) have suggested that covert rehearsal is more efficient than overt rehearsal because the former can be carried out at a faster rate and with words overlapping in time. If this is the case, then requiring overt rehearsal might reduce the amount of information participants can rehearse, and performance in an overt rehearsal condition might show poorer recall compared to conditions without this requirement. The one study that has investigated this issue so far (Tan and Ward, 2008) found no support for this conjecture: They observed similar recall performance for overt-rehearsal and silent groups. Given the scant research on this area, we also included groups without an overt rehearsal instruction (Silent groups) in our study.

There were two additional manipulations in the present study. First, in order to examine the impact of the non-verbal distractor processing on rehearsals, Experiment 1 compared spontaneous rehearsals in simple and complex span. Second, we also included a manipulation of presentation rate. Tan and Ward (2008) showed that both the frequency of cumulative rehearsals and recall accuracy increased with slower presentation rates (see also Souza & Oberauer, 2018). We aimed at replicating these patterns in our simple span groups, and to extend these results to complex span. In complex span, the temporal gaps between memory items are filled with distractor activity, so extending these gaps (i.e., slowing the presentation rate of memory items) should enable more cumulative rehearsal only to the extent that the distractor task does not disrupt rehearsal, and it should improve recall only to the extent that the distractor task does not disrupt those processes that, in simple span, lead to better recall at slower presentation rates. We also assessed whether the beneficial effects of a slower presentation rate were retained when participants were required to recall the studied words in a delayed test. Previous research (Souza & Oberauer, 2017) showed that slow rates improves recall both over the short-term and the long-term. We assessed whether this holds in our study.

### Method

#### Design

Experiment 1 consisted of a 2 × 2 × 2 mixed-design consisting of span task (simple span vs. complex span), rehearsal instruction (overt vs. silent) – manipulated between-subjects – and presentation rate (medium vs. slow) – which was manipulated within-subjects.

#### Participants

Eighty students of the University of Zurich (50 women; average age = 25 years) participated in exchange for 15 Swiss francs or course credit. Participants were native German speakers. They signed an informed consent form prior to the study and they were debriefed at the end. The study protocol is in line with the guidelines of the institutional ethics review board. Participants were randomly assigned to one of four groups (n = 20), which differed regarding the type of span task (Simple Span or Complex Span) and rehearsal instruction (Silent or Overt rehearsal).

#### Materials and Procedure

Lists of six words were presented for forward serial recall. Words appeared one by one in the center of the screen for 1 s, followed then by a 1.22-s or 4.22-s gap, yielding presentation rates of 2.22 s and 5.22 s per word, respectively. These intervals correspond roughly to the medium and slow presentations rates used by Tan and Ward (2008), and we are going to refer to these as the medium and slow presentation rate conditions, respectively.

**Figure 1:**
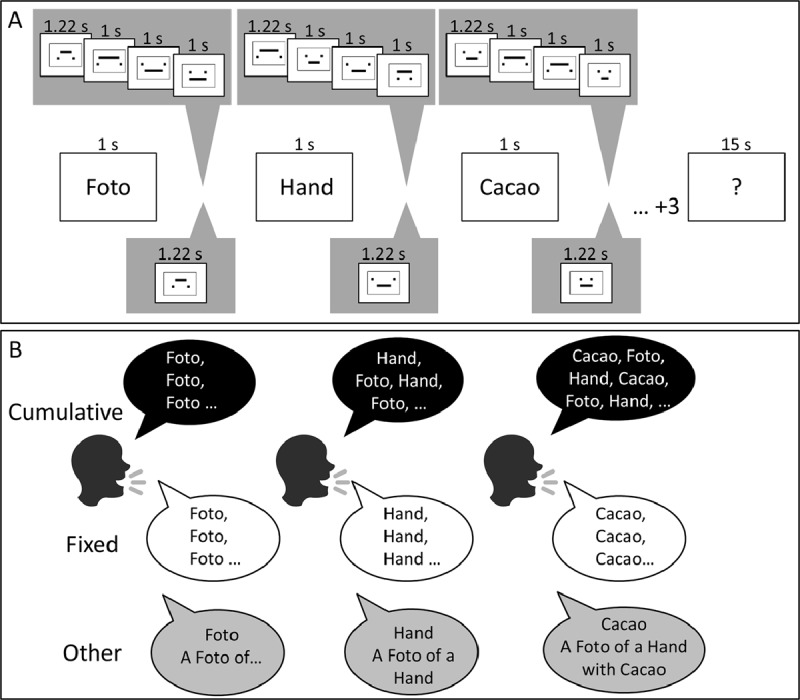
**Panel A.** Illustration of the flow of events in complex span trials with a slow (5.22 s between memory items, top row) and medium presentation-rate (2.22 s between memory items, bottom row). **Panel B** illustrates the classification of hypothetical rehearsal patterns into cumulative rehearsals, fixed rehearsals, and other.

During the gaps between words, the complex span groups worked on a spatial distractor task (described below; see Figure 1A), whereas the simple span groups were shown a blank screen. Lists with short and long gaps strictly alternated between trials (order counterbalanced across participants). The word lists on each trial were drawn from a pool of 625 German nouns, 4–5 letters long. We created 20 sets of 48 six-word lists. Each set was used for one participant in each experimental group.

Each trial started with a message indicating whether the upcoming trial will have short or long gaps between words. Participants self-initiated the presentation of the list by pressing the spacebar. Participants in the *Overt rehearsal* groups were instructed to say aloud the just presented word and any of the words they were rehearsing during the study phase; whereas participants of the *Silent* groups were instructed to read the words aloud and remain silent otherwise. These instructions were modelled closely after Tan and Ward (2008). At the end of the list, a question mark and a tone signaled the start of the recall phase (see Figure 1A). Participants orally recalled the items in forward serial order, saying “blank” for not remembered items. After 15 s, a blank screen and a tone indicated the end of the recall phase, which was followed by a 2-s inter-trial interval. Rehearsals and recalls were recorded by the computer and later coded by the experimenter.

The complex span groups performed a spatial decision task (see Figure 1A) during the gaps between words: a horizontal line was displayed above or below a pair of aligned squares (Vergauwe, Barrouillet, & Camos, 2010). The task was to decide whether the line fits the space between the squares by pressing the left-arrow key for a yes; and the right-arrow key for a no response. We chose this task because it requires no articulation and little involvement of verbal representations in general. If there are two independent maintenance processes (Camos et al., 2009), refreshing and rehearsal, then this task should disrupt refreshing by virtue of capturing central attention, but not disrupt rehearsal.

The short- and long-gap inter-word intervals (aka. medium and slow conditions) were filled with one and four spatial decisions, respectively. Participants had a limited time to perform each decision. The available time per distractor operation was selected such that the cognitive load was held constant between the two presentation rate conditions. Following Barrouillet et al. (2004), the cognitive load is defined as the ratio between the amount of time the distractor task diverts attention away from the memory items to the total retention interval. In the medium presentation rate, participants had 1.22 s to perform the single spatial decision presented in the short gap between words; whereas in the slow presentation rate condition, participants had 1.22 s for the first decision, and 1 s for each subsequent decision presented during the long gap between words. These times were previously found to equate cognitive load between these conditions (Oberauer & Lewandowsky, 2014). If participants did not respond within this time-limit, the stimulus was removed from the screen, a time-out error was logged, and the next stimulus was presented. There was a 5-min practice phase before the actual experiment in which participants in the complex span groups completed 250 spatial decisions to get acquainted with the response time limits.

At the end of the experiment, participants were given a blank sheet of paper and asked to write down any words they remembered learning during the experiment (delayed free recall test). Next, they filled in a post-experimental questionnaire, and were debriefed regarding the purpose of the experiment.

#### Data analysis

We analyzed the data of all our experiments with Bayesian mixed effect models (LME) using the lmBF function from the BayesFactor package (Morey & Rouder, 2015) implemented in R (R core team, 2017). This function computes the strength of the evidence for the specified model (M_1_) against a Null model (M_0_). The ratio of the likelihood of these two models is the Bayes factor (BF_10_). The BF should be interpreted as the multiplicative factor by which our ratio of prior beliefs in the two models should be updated in light of the data. BFs below 3 are usually regarded as “weak evidence”; BFs between 3 and 10 are regarded as providing “substantial evidence”; BFs between 10 and 100, as providing “strong evidence”; and above 100, “decisive evidence” in favor of one model over the other (Kass & Raftery, 1995). The BF for the Null model over the alternative (i.e., BF_01_) can be computed as 1/BF_10_, and they should be interpreted in the same way.

We specified a set of models, starting from a full model with all of our predictors and interactions thereof. Across several steps, we assessed the evidence for each fixed predictor by dropping this term from the full model. By computing the ratio of the BF_10_ for the model including the effect of interest with the BF_10_ of the model excluding it, we obtained the strenght of the evidence supporting the effect of interest in the data. When this ratio was below 1, we removed the term from the full model – meaning that this term was excluded from the full model that we subsequently used to assess the evidence for all remaining predictors. When the ratio was above 1, the term was retained in the full model. We repeated these steps until we tested the evidence for all predictors. We always started by assessing the evidence for the interaction terms and moved up to the main effects. All models included a random intercept for participant, and random slopes over participants for the effects of the variables manipulated within-subjects (Barr, Levy, Scheepers, & Tily, 2013).

The materials, data, and analysis scripts for the experiments reported in this paper are available on the Open Science Framework: https://osf.io/wu3mc/.

### Results

#### Distractor Processing in Complex Span

We excluded one participant from the Overt Complex Span group due to chance performance in the spatial distractor task (Proportion Correct *M* = 0.43), leaving this group with N = 19. Table 1 presents the average performance in the distractor task across the two presentation rates and rehearsal groups. We compared performance of the two groups in each presentation rate with Bayesian *t*-tests. Participants in the Overt Rehearsal Complex Span group tended to respond slightly less accurately and more slowly than participants in the Silent Complex Span group, but there was not enough evidence in the data to support a difference between groups (i.e., the BF was ambiguous). We also computed the CL in each condition by summing up the RTs over all processing episodes following each word and dividing this sum by the total duration of the processing phase (1.22 s for the medium rate; 4.22 s for the slow rate; see CL in Table 1). The values of the CL were around 0.5, which was the value we aimed for.

**Table 1:** Performance in the Spatial Distractor Task for the Complex Span Groups in Experiment 1. Values in Bold Indicate the Evidence (Bayes Factor, BF) for the Alternative Hypothesis over the Null for the Difference in Performance Between the Two Groups.

Rate	Rehearsal		Proportion Correct		Reaction Time (s)		CL

			M	95% CI	M	95% CI	

Medium	Silent		0.94	[0.93, 0.95]	0.622	[0.616, 0.628]	0.51
	Overt		0.92	[0.91, 0.93]	0.647	[0.640, 0.655]	0.53
		**BF_10_**	**1.17**		**0.74**		**0.73**
Slow	Silent		0.91	[0.90, 0.92]	0.583	[0.580, 0.587]	0.54
	Overt		0.89	[0.88, 0.90]	0.604	[0.600, 0.609]	0.55
		**BF_10_**	**0.70**		**0.98**		**0.44**

*Note*: CI = 95% within-subjects confidence interval. CL = cognitive load.

#### Immediate Recall

**Figure 2:**
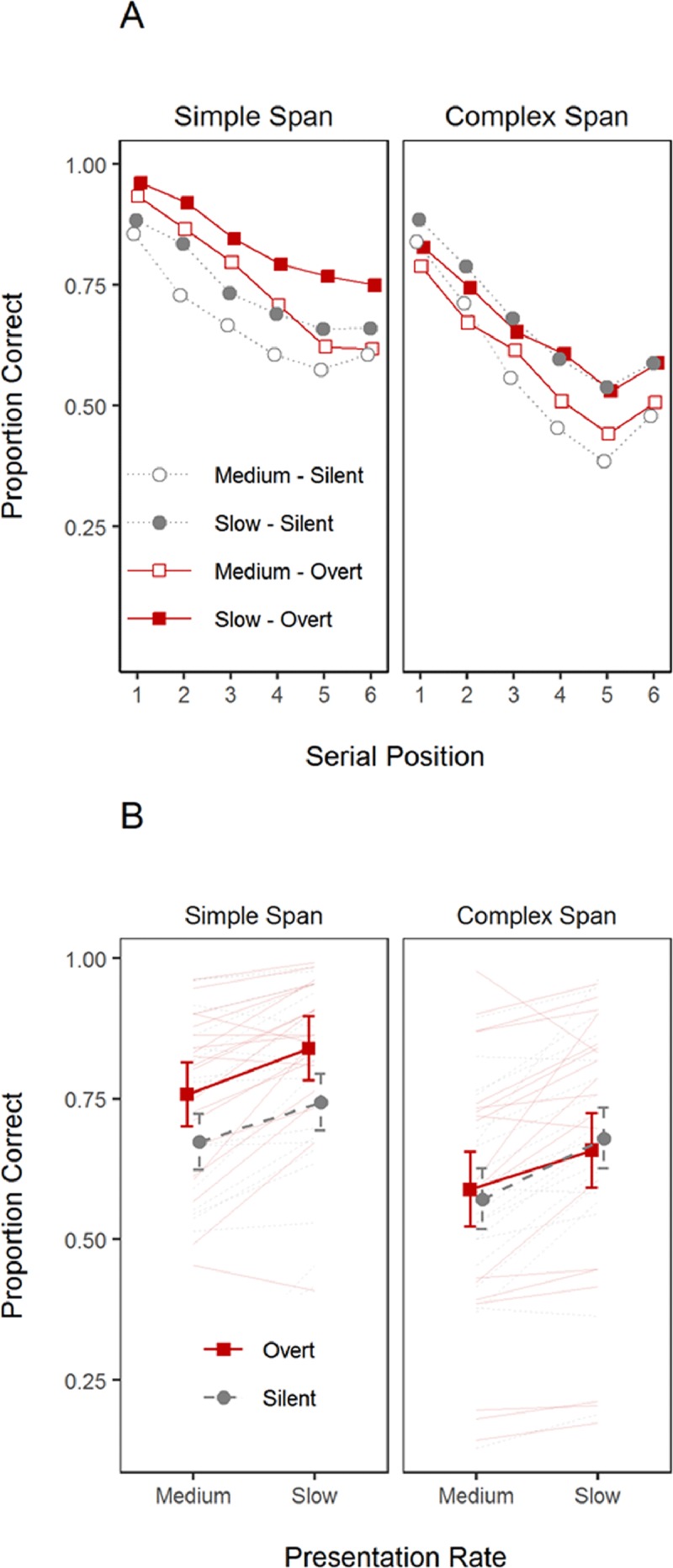
**Panel A.** Serial position curves for each span task and rehearsal instructions (between-subject variables), and presentation rate (within-subject variable) in Experiment 1. **Panel B** presents overall proportion of correct responses as a function of presentation rate, task, and rehearsal instruction. Faint lines represent individual participants. Error-bars correspond to the standard deviation.

Recall was scored as the proportion of items reported in the correct serial position. Figure 2A presents serial position curves for each experimental group as a function of presentation rate. Given that we were not interested in the main effect of span task (manipulated between-subjects), we analyzed the data of the two span tasks separately in order to keep the number of predictors and interactions in our models within a reasonable range. For each task (Simple Span or Complex Span), we specified models having as fixed predictors Rehearsal (silent vs. overt), Rate (medium vs. slow), Serial Position (1–6), and their interactions. All of these variables were coded as categorical predictors. Table 2 lists the evidence (BF_10_) for inclusion of each term in the modeling of the data.

**Table 2:** Bayes Factors Quantifying the Strength of Evidence for the Main Effects and Interactions of the Variables Manipulated in Experiment 1 on Serial-Recall Accuracy.

Term	Simple Span	Complex Span

Overt Rehearsal Instruction	1.06	0.56
Rate	84’650	91’111
Serial Position	7.53 × 10^29^	1.05 × 10^32^
Overt Rehearsal × Rate	0.25	0.59
Overt Rehearsal × Serial Position	0.11	0.21
Rate × Serial Position	2.14	1.01
Three-way interaction	0.66	0.06

*Note*: The data of each span task was analyzed with different sets of models.

As shown in Table 2, the requirement to perform overt rehearsal had a benign effect. In Simple Span, the BF_10_ was indecisive about whether or not there was an overt rehearsal effect. Numerically, the Simple Span Overt group recalled more words (see also Figure 2B), but in light of the weak BF, this difference might be due to sampling noise. The ambiguous BF we observed for the Overt Rehearsal effect does not address the question of whether overt rehearsal is less efficient than covert rehearsal, which predicts worse recall in the overt rehearsal condition. To directly assess this possibility, we compared performance of the silent and overt groups in each span task with Bayesian one-sided *t*-tests, testing the prediction that the Silent group performs *better* than the Overt group. There was substantial evidence against a negative effect of overt rehearsal in Simple Span (Medium rate BF_10_ = 0.15; Slow rate BF_10_ = 0.13). The evidence was only tentative in Complex Span (Medium rate BF_10_ = 0.27; Slow rate BF_10_ = 0.39).

Presentation rate strongly impacted serial recall: recall in simple and complex span was higher with slow than with medium presentation rate (see Figure 2A and Table 2). There was also overwhelming evidence for an effect of serial position in both tasks, reflecting the typical findings of strong primacy and small recency observed in immediate serial recall tasks. There was no evidence to support any interaction involving the overt rehearsal instruction. The only interaction term that received at least ambiguous support was that of presentation rate with serial position: serial position curves tended to be less steep in the slow presentation rate than in the medium one.

#### Rehearsal

For the Overt rehearsal groups, we coded the utterances occurring during each gap following a memory item (regardless of their correctness). In a first step, we counted the total number of words rehearsed in each trial by summing up the number of uttered words across all time gaps. We then compared this total between the two presentation rates and the two overt rehearsal groups. Participants rehearsed about 11 words in the medium presentation rate (Simple Span *M* = 11.2, [95% CI: 9.1, 13.4]; Complex Span *M* = 10.9 [7.4, 14.4]), and about 19 words in the slow presentation rate (Simple Span *M* = 19.2, [17.0, 21.4]; Complex Span *M* = 18.7 [15.2, 22.2]). There was overwhelming evidence for the difference between presentation rates in terms of the number of rehearsed words (BF_10_ = 10’873). Comparison between span tasks, however, yielded somewhat ambiguous evidence for the Null (BF_10_ = 0.58) and for an interaction (BF_10_ = 0.43).

In the second step, we analyzed the pattern of words uttered in each gap in order to classify them in terms of the strategies used by participants. First of all, we created a category called *Silence* which was registered when no word was spoken. When participants did speak words during the list presentation, we categorized the rehearsal patterns in one of four categories following closely the schema used by Tan and Ward (2008). For illustration of these rehearsal patterns see Figure 1B.

1. A *Fixed* strategy was coded when participants simply read the words as they were presented; or when they repeated the currently presented word multiple times, but no other word was rehearsed in that time gap.
2. A *Cumulative* strategy was coded when somewhere during the gap participants rehearsed all list items presented so far in their correct serial positions. For example, at gap 4, rehearsing the word sequence 1-2-3-4 somewhere during the gap was deemed cumulative (e.g., 1-1-2-3-4, 4-4-1-2-3-4).
3. A *Partial Cumulative* strategy was coded when somewhere during the gap participants started rehearsing from the beginning of the list and proceeded in forward order, but did not manage to rehearse all presented items (e.g., 4-4-1; 4-4-1-2; 4-3-1-2).
4. *Other* was used for any other type of strategy. Most of the patterns that fell into the latter category comprised rehearsal of pairs or triplets of items (e.g., 4-3-4; 2-3-3-4).

**Figure 3:**
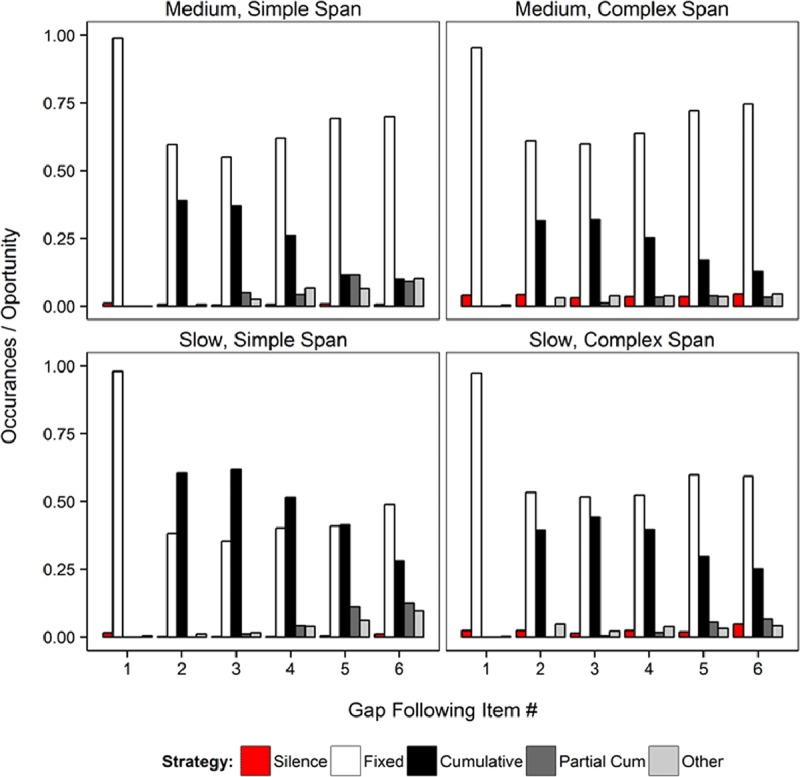
Relative frequency of each type of rehearsal strategy plotted separately for the overt simple span and overt complex span groups, and the medium and slow presentation rates in Experiment 1.

Figure 3 presents the proportion of times participants used one of the rehearsal strategies described above in the gap immediately following presentation of list items 1 to 6. Figure 3 shows, first, that the most prevalent strategy was to simply read the current presented item (Fixed strategy). This is not surprising given that participants were explicitly instructed to read all presented items aloud, such that, if they merely complied with this instruction, their verbal output would be classified in the Fixed category. The only exception to the dominance of the Fixed category was the slow presentation rate for the Overt Simple Span group, in which cumulative rehearsal was the most frequent strategy. Second, when participants rehearsed – over and above following the instruction to read the presented items – then their most common choice was to rehearse items in a cumulative fashion. As the list-length increased, though, a full cumulative strategy became increasingly more difficult to sustain, which led participants to perform partial cumulative rehearsal attempts. Other types of rehearsal strategies occurred in a very small proportion of trials, and only towards the end of the list. Third, cumulative rehearsal became the dominant strategy in simple span when a slow presentation rate was used. For complex span, the slow presentation rate did not lead to such a pronounced increase of cumulative rehearsal.

To provide quantitative evidence for the difference in rehearsal patterns between the simple and complex span groups, we submitted the frequencies of rehearsal patterns to a Bayesian Contingency Table test (Gunel & Dickey, 1974) using the test available in the BayesFactor package. For this analysis, we first excluded responses in gap 1 because people could not do anything but fixed rehearsal at this time point. Second, we excluded the categories of silence and partial cumulative attempts, because they were very rare, and of minor theoretical interest. Lastly, we summed the strategies across serial positions 2–6 to get a total count of strategy use. This allowed us to compare the frequencies of three categories (Fixed, Cumulative, and Other) between the two tasks. There was overwhelming evidence against a difference in rehearsal patterns between span tasks in the medium rate (BF_10_ = 3.7 × 10^–6^), but overwhelming evidence for a difference in the slow rate condition (BF_10_ = 2.43 × 10^10^). This reflects the fact that participants were much more likely to rehearse cumulatively in simple span in the slow rate, whereas fixed rehearsals were more frequent in complex span.

#### Rehearsal × Recall

**Figure 4:**
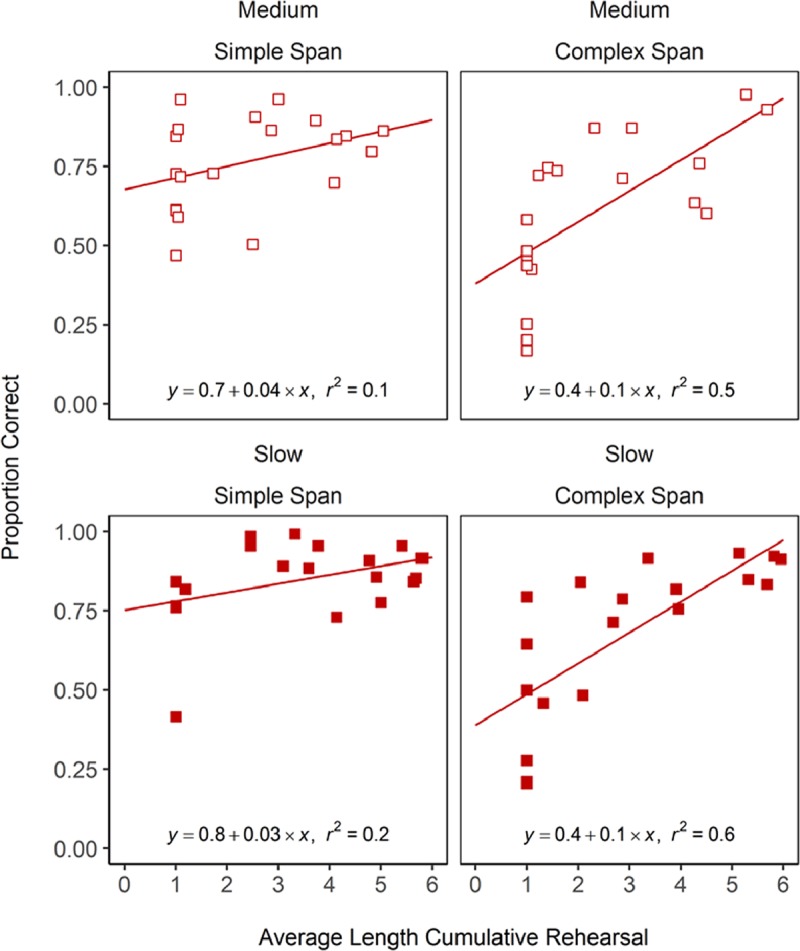
Scatterplots showing the relationship between cumulative rehearsal-sequence length against recall accuracy across participants in Experiment 1. The line and coefficients obtained when fitting a regression line to this data are shown inside each panel.

Following Tan and Ward (2008), we computed the maximum length of the rehearsal-sequence in a given trial and correlated this measure with overall recall accuracy in each presentation rate condition (see Table 3). For example, if in trial 1, the participant rehearsed at most items 1 to 5 in the correct order, then the assigned maximum rehearsal length was 5. Non-cumulative rehearsals and fixed rehearsal were assigned a length of 1. We also plotted the degree of cumulative rehearsal used by an individual against their overall recall accuracy in each condition (see Figure 4).

**Table 3:** Correlation Coefficients Between the Average Maximum Length of the Rehearsal Sequence and Recall Accuracy.

Experiment	Group	Rate/Block	Pearson R

1	Simple Span	Medium	0.373
		Slow	0.394
1	Complex Span	Medium	0.672
		Slow	0.748

Table 3 and Figure 4 show a positive relation between the degree of cumulative rehearsal and recall for both groups and both presentation rates. The correlation between recall and rehearsal, however, was smaller in simple span than in complex span. There was ambiguous evidence for cumulative-rehearsal length predicting overall levels of recall accuracy in simple span (medium rate, BF_10_ = 1; slow rate, BF_10_ = 1.2). The evidence for this relationship was strong in complex span though (medium rate, BF_10_ = 20.4; slow rate, BF_10_ = 97.3).

#### Delayed Free Recall

**Figure 5:**
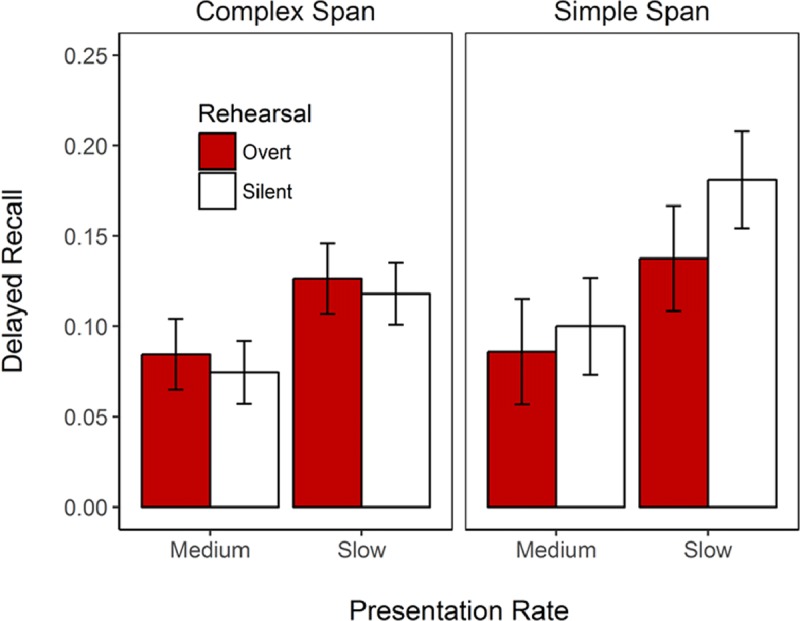
Proportion of words studied during fast and slow rate trials that were recalled in the surprise free recall test at the end of Experiment 1. Error bars represent 95% within-subjects confidence intervals.

Participants completed a surprise delayed free-recall test at the end of the experiment in which they were asked to write down any words they remembered learning during the experiment. We computed the proportion of recalled words that were studied in the fast and slow rate conditions (see Figure 5). Participants recalled more words learned during the slow rate trials than from the fast rate trials, and the best model of this data only included the main effect of presentation rate (BF_10_ = 3.9 × 10^6^). There was ambiguous evidence against a main effect of span task (BF_10_ = 0.69) and of overt rehearsal (BF_10_ = 0.39).

### Discussion

We used for the first time the overt rehearsal protocol to examine rehearsal in complex span as well as simple span. The overt rehearsal protocol allows for the direct observation of when and how participants rehearse. Despite concerns that overt rehearsal protocols could be disruptive to performance (Cowan & Vergauwe, 2015), the comparison between the overt-rehearsal groups and silence groups yielded no support for worse performance in the overt rehearsal groups (see also Tan & Ward, 2008) (but see Murray, 1967 for one study where costs were found).

Our main goal in Experiment 1 was to observe when and how participants rehearse in complex span, and to assess whether rehearsal frequency and rehearsal patterns were similar across complex span and simple span. We will discuss next our findings regarding rehearsal frequency, rehearsal strategies, the slow-rate benefit, and the relationship between rehearsal and recall.

#### Rehearsal Frequency

The number of rehearsed words was similar between span tasks. This result is in line with studies of self-reported memory strategies, which indicate a high prevalence of articulatory rehearsal in both simple and complex span (Bailey, Dunlosky, & Hertzog, 2009; Bailey, Dunlosky, & Kane, 2008; Bailey et al., 2011; Dunlosky & Kane, 2007). Our study extends this finding by providing direct evidence that the number of uttered words and the selection of rehearsal schedules (fixed, cumulative, partial cumulative, and other types) are similar across these tasks. Furthermore, these results indicate that the processing of a non-verbal distractor task does not compete with articulating words.

#### Rehearsal Strategies and Cumulative Rehearsal

Although the number of uttered words did not differ between span tasks, the relative frequency of use of different rehearsal schedules was changed by distractor processing. Critically, cumulative rehearsal was substantially more frequent in simple span than complex span. This finding can be interpreted in two ways. One explanation is that cumulative rehearsal is disrupted by the requirement to concurrently perform a non-articulatory distractor task. This explanation would imply a departure from the popular assumption that articulatory rehearsal is an attention cost-free mechanism and, therefore, independent of the cognitive load imposed by the distractor task (e.g., Vergauwe et al., 2014). In simple and complex span tasks, the rehearsal schedule needs to be updated after presentation of each new word. Implementing a new rehearsal schedule does require attention, albeit to a small degree (Guttentag, 1984; Thalmann, Souza, & Oberauer, 2019), which is in line with evidence that rehearsal is a process that involves frontal brain activity (Awh et al., 1996). Carrying out the distractor task in complex span might interfere with updating the rehearsal schedule for cumulative rehearsal – thereby reducing its frequency. In contrast, the fixed rehearsal strategy was already set-up because participants were asked to read the presented words aloud and they started with this routine prior to the onset of the distractor task. Another explanation for this difference is that rehearsal is not the cause of good memory but an effect of it: One needs to remember the memory list in order to rehearse it. Given that distractor processing reduces the strength of the memory trace in WM, previously presented words will be less accessible for retrieval and for rehearsal.

#### Slow-Rate Benefit

Experiment 1 replicated the finding of better short-term and long-term memory with slower presentation rates (Souza & Oberauer, 2017, 2018; Tan & Ward, 2008), even when the gap between words were filled with a distractor task engaging central attention (Oberauer & Lewandowsky, 2014). We have no ready explanation for this slow-rate benefit. First, it cannot be attributed to rehearsal because the slow-rate benefit was of about equal size for all serial positions despite the fact that items from the beginning of the list were rehearsed much more often. Moreover, slow presentations rates have been found to benefit serial recall even under AS (Longoni, Richardson, & Aiello, 1993; Souza & Oberauer, 2018). Second, the benefit of slow presentation cannot be attributed to the additional time people can devote attention to each list item, because the size of the benefit was about the same in simple and complex span. In the complex span task, distractor processing occupied attention for a substantial amount of time, so that any beneficial effect of paying attention to the memory representations should have been weaker in complex span than in simple span. Therefore, the slow-presentation benefit is not easily explained by attention-demanding processes such as refreshing (Barrouillet, Portrat, & Camos, 2011; Camos et al., 2018) or even consolidation (Bayliss, Bogdanovs, & Jarrold, 2015; Ricker, Nieuwenstein, Bayliss, & Barrouillet, 2018). Third, a possible explanation of the slow-presentation benefit in terms of temporal distinctiveness seems also rather unlikely because temporal distinctiveness does not benefit performance in serial recall tasks (Lewandowsky, Brown, Wright, & Nimmo, 2006; Nimmo & Lewandowsky, 2005, 2006). One possible explanation left is related to the use of elaborative rehearsal: it is possible that the long inter-item interval favored the use of more effective maintenance strategies. In line with that possibility, Souza and Oberauer (2018) observed larger slow-rate benefit for concrete than abstract words. We note, however, that it is still unclear whether elaboration could improve performance in WM tasks, with some studies finding no evidence for a benefit (Bartsch, Loaiza, Jäncke, Oberauer, & Lewis-Peacock, 2019; Bartsch, Singmann, & Oberauer, 2018). These studies provided opportunities for elaboration after all items were encoded though, and not directly after presentation. Hence future studies will have to examine whether and how elaboration may contribute to the slow-rate effect.

#### Cumulative Rehearsal vs. Recall

We observed a positive correlation between recall accuracy and the length of cumulative rehearsal. The evidence for such a relation was very strong in complex span, but weak in simple span. Albeit the evidence supporting the correlation in simple span was weaker, the correlation coefficients were close to the one obtained in the meta-analysis by Souza and Oberauer (2018).

The existence of a positive correlation between rehearsal and recall, however, is not sufficient to establish that rehearsal is beneficial to performance. In order to probe for the existence of a causal link between these variables, one needs not only to observe rehearsal but also to manipulate it. Overt rehearsal protocols can be a useful tool in this case as well: They allow one to measure the effectiveness of the manipulations assumed to affect rehearsal (e.g., of an instruction to rehearse in forward cumulative fashion) and relate rehearsal to recall. This was the main goal of Experiment 2.

## Experiment 2

Experiment 2 assessed the causal relationship between cumulative rehearsal and serial recall in complex span with a manipulation of rehearsal instruction. We were concerned, however, that merely instructing participants to engage in cumulative rehearsal might over-tax their ability, as it effectively imposes a triple-task demand (i.e., encode words, process the distractor task, and rehearse in cumulative order). To circumvent this problem, we introduced a Training block in which we instructed participants to perform cumulative rehearsal without having the competition of the spatial-fit task (i.e., during simple span). We reasoned that training cumulative rehearsal in a simplified setting would provide the best chance of participants successfully applying a cumulative rehearsal strategy in complex span trials, and thereby arguably benefiting from it. We exposed participants to the complex span task before (Pre-test) and after this rehearsal-training block (Test) with the goal of assessing changes in rehearsal patterns and recall accuracy due to cumulative rehearsal. A control group was exposed to the same order of experimental blocks (i.e., Pre-test, Training, and Test) with the only difference that they did not receive the cumulative rehearsal instruction in the Training block. Given that the slow presentation rate provided the most opportunity for rehearsal, Experiment 2 only included the slow rate condition.

### Method

#### Design

Experiment 2 consisted of 2 × 3 mixed-design having Rehearsal instruction (Control vs. Cumulative Rehearsal) manipulated between-subjects, and Experimental Block (Pre-Test, Training, Test), which was manipulated within-subjects.

#### Participants

Sixty-three students of the University of Zurich took part in a single long session (lasting 1.5 hs) in exchange for course credit or a reimbursement of 23 Swiss francs. Participants were randomly assigned to one of two groups: a *Control group* (*N* = 32), and a *Cumulative Rehearsal group* (*N* = 31). Five participants in the Control group performed the distractor task in complex span with low accuracy (Proportion Correct, *M* = 0.59, 0.60, 0.53, 0.61, and 0.05; the latter showed low accuracy due to time-outs), and were excluded from the final analyses, leaving a total sample of *N* = 27 in this group. Three participants from the Cumulative Rehearsal group were excluded: two because they had poor accuracy in the distractor task (*M* = 0.06 and 0.22^1^), and one because they misunderstood the rehearsal instructions (as indicating that Cumulative Rehearsals were to be avoided). The final sample of this group was *N* = 28.

#### Materials and Procedure

We used the two word-sets employed by Souza and Oberauer (2018). One set consisted of words with high ratings of concreteness and imageability whereas the other set consisted of words with low ratings on both dimensions, which were selected from the “Semantischer Atlas” data base (Schwibbe, n.d.). Both sets were equated in terms of word length (mean = 7.8 characters) and word frequency (mean log frequency among 4.5 million words = 4.9). Half of the lists in each condition were sampled from each set, and word lists sampled from these two sets strictly alternated in the experiment (with order counterbalanced across participants).

The experiment comprised three blocks of trials which were presented following an ABA design (Pre-test, Training, and Test): in the Pre-test and Test blocks, participants completed trials of complex span, whereas the Training block consisted of trials of simple span. As in Experiment 1, complex span trials comprised the presentation of a word (1 s onscreen) followed by a sequence of four spatial-fit distractor decisions to be completed during a 4.22 s window (i.e., the slow rate condition). Each trial comprised 6 word-distractor processing episodes. At the end of the last episode, participants were prompted to recall all words in forward serial order by typing the first three letters of each word. In simple span trials, a sequence of 6 words (1 s onscreen; 4.22 s blank) was presented, followed by the forward serial recall test. Participants completed 20 trials in each experimental block.

As in Experiment 1, all participants completed a practice phase with the spatial-fit decision task (250 trials performed in a 5-min warm-up phase) before entering the experiment proper. All participants were instructed to rehearse aloud in case they were using this strategy to maintain the words, and they were informed that their rehearsals would be recorded. Unlike Experiment 1, participants were not required to read the words aloud upon their presentation. This allowed us to measure their natural engagement in the fixed rehearsal strategy, which was not possible in Experiment 1 due to the instruction to read the words aloud.

The two experimental groups differed regarding the rehearsal instructions that were presented prior to the start of the Training block (i.e., during simple span trials). Participants in the control group were simply instructed that the requirement to perform the spatial-fit task will be removed. Participants in the Cumulative Rehearsal group were additionally instructed to rehearse all words presented in cumulative forward order, and they were presented with examples of the cumulative rehearsal strategy. After the simple span block, all participants completed a new set of complex span trials (Test block). Participants in the control group received the same standard instructions on how to complete complex span trials as in the first block. Participants in the cumulative rehearsal group were further instructed to continue using the cumulative rehearsal strategy they had practiced in the prior block.

### Results

#### Distractor Processing

Due to a programming error, accuracy in the distractor task was not recorded for the first three participants in the experiment (two participants from the Control group and one participant from the Cumulative Rehearsal group). We removed the data of these participants from analysis of the distractor task performance, but retained it for the main analyses. One participant of the Cumulative Rehearsal group reversed the response mapping (responded with the left button to misfit instead of using the right button), but otherwise performed the task with sufficiently high accuracy (i.e., with accuracy above 0.7), and their data were retained for further analyses.

Table 4 presents performance in the spatial fit task and provides the BF for the group comparisons in each condition. As shown in Table 4, average performance tended to be similar between groups, even after the introduction of the rehearsal instruction.

**Table 4:** Performance on the Spatial Fit Task Used as Distractor Task in the Complex Span Blocks in Experiment 2 and Evidence (BF) for Group Differences.

Block	Group	Proportion Correct		Reaction Time (s)	

		M	95% CI	M	95% CI

**Pre-test**	Control	0.86	[0.83, 0.90]	0.609	[0.581, 0.636]
	Cumulative Reh.	0.89	[0.87, 0.91]	0.601	[0.580, 0.622]
	**BF_10_**	**0.69**		**0.30**	
**Test**	Control	0.89	[0.86, 0.91]	0.585	[0.562, 0.609]
	Cumulative Reh.	0.89	[0.86, 0.92]	0.599	[0.578, 0.620]
	**BF_10_**	**0.28**		**0.39**	

*Note*: CI = 95% between-subjects confidence interval.

#### Rehearsal

Table 5 presents the average number of rehearsed words in each block per group, and the evidence for between-group differences in each experimental block, as well as intra-group differences as a function of Block (i.e., comparing Pre-test vs. Test). The number of rehearsed words did not differ between the two groups in the Pre-test. Instructing participants to rehearse cumulatively during simple span doubled the number of rehearsals in the Cumulative Rehearsal group compared to the Control group in the Training block. This increase in rehearsals in the experimental group was retained in the Test block when participants were re-exposed to complex span.

**Table 5:** Average Number of Rehearsed Words in Each Experimental Block in Experiment 2. The Evidence for the Difference in Rehearsal Between the Groups and the Blocks were Assessed with Bayesian T-Tests.

Block	Control		Cumulative Rehearsal		Group

	M	95% CI	M	95% CI	BF_10_

Pre-test	20.4	[14.5, 26.3]	21.8	[17.4, 26.2]	**0.29**
Training	18.3	[13.4, 23.2]	38.1	[34.4, 41.8]	**5.9** × **10^5^**
Test	18.3	[12.4, 24.2]	36.9	[32.4, 41.5]	**3718**
**Pre-test vs. Test – BF_10_**	**0.34**		**4762.5**		

*Note*: CI = 95% within-subjects confidence interval.

**Figure 6:**
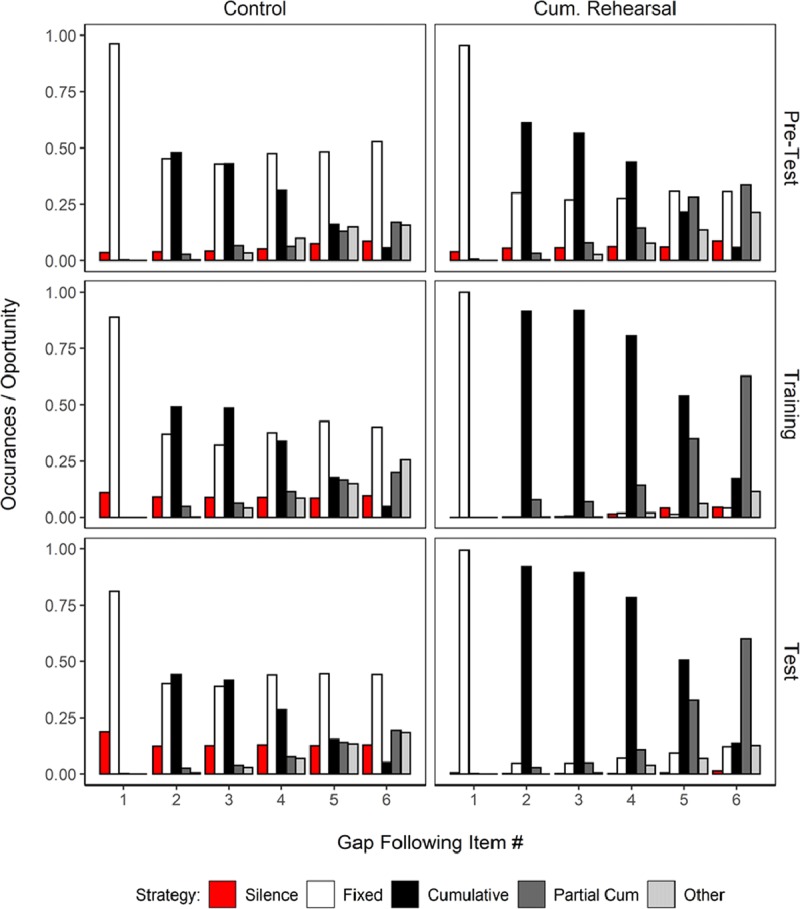
Relative frequency of each type of rehearsal strategy plotted separately for the three experimental blocks (Pre-test, Training, and Test) and two groups (Control vs Cumulative Rehearsal) in Experiment 2.

Figure 6 shows the frequency of each rehearsal strategy. Fixed and cumulative rehearsals were relatively frequent in the Pre-test block. Given that only trials with slow presentation rate were used, this result replicates the finding of Experiment 1: long gaps between words are used to rehearse the memoranda, and rehearsals tend to be performed in forward order. Furthermore, participants tended to remain silent in a larger proportion of the trials than observed in Experiment 1. This increase in silence entries is to be expected given that we removed the requirement to read the words aloud. It is also in line with studies assessing self-reported strategies that indicate that rehearsal is not used in every trial.

The Cumulative Rehearsal group showed somewhat larger proportions of cumulative rehearsals compared to the Control group in the Pre-test. A Bayesian Contingency Table Test (having the Fixed, Cumulative, and Other categories collapsed across all time gaps, except gap 1, as done in Experiment 1) yielded overwhelming evidence for a difference between groups in rehearsal patterns (BF_10_ = 3.4 × 10^22^).

Figure 6 also shows that the instruction to perform cumulative rehearsals provided in the Training block accentuated the use of cumulative rehearsals in the Cumulative Rehearsal group compared to the Control group, and the difference in strategy-use between groups persisted (BF_10_ = 4.8 × 10^339^). The difference between groups in the use of cumulative rehearsals remained in the Test block (BF_10_ = 4.4 × 10^253^). Because the Cumulative Rehearsal group already used a cumulative rehearsal strategy more often than the Control group in the Pre-test, it is more informative to compare the change in patterns of rehearsals across the Pre-test and Test blocks to assess for the effect of our rehearsal instruction: The Cumulative Rehearsal group showed a substantial increase in cumulative rehearsals (BF_10_ = 2.1 × 10^126^), but the Control group did not (BF_10_ < 0.0001).

We also computed the maximum cumulative rehearsal length in the Pre-test and Test blocks for the two groups. The cumulative rehearsal-length did not differ between these blocks for the Control group (Pre-test, *M* = 2.53 [95% *CI*: 2.29, 2.77]; Test, *M* = 2.47, [2.27, 2.67]), BF_10_ = 0.21. There was overwhelming evidence for an increase in the cumulative rehearsal length for the Cumulative Rehearsal group (Pre-test, *M* = 3.08 [2.70, 3.46]; Test, *M* = 4.35 [4.10, 4.60]), BF_10_ = 3546. Moreover, in the Pre-test, the average cumulative rehearsal length did not substantially differ between groups (BF_10_ = 0.68), but this difference was substantial in the Test block, BF_10_ = 19728. Hence, the instruction to perform cumulative rehearsals did increase the frequency and reach of this strategy.

**Figure 7:**
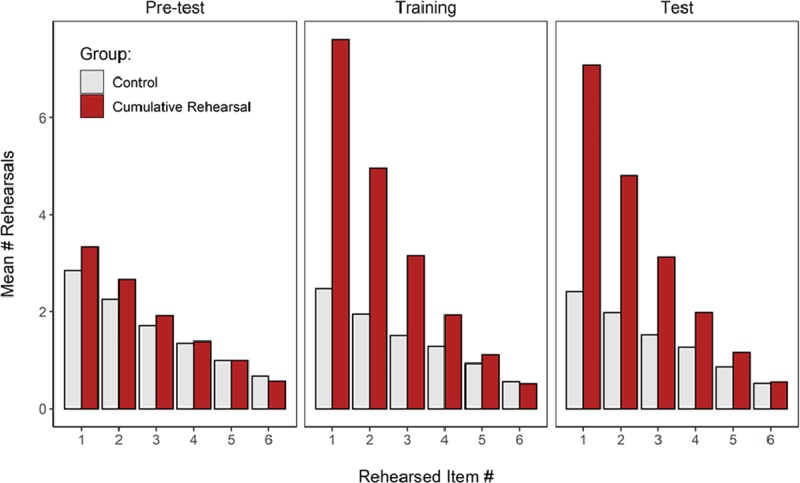
Average frequency of rehearsals of each list item during the trial plotted separately for the three experimental blocks of Experiment 2, and the two groups.

Figure 7 shows the average rehearsal frequency for words in each serial position. For both groups and in all experimental blocks, earlier list items received most rehearsals, as would be expected from cumulative rehearsal being a very prevalent strategy. The instruction to rehearse cumulatively provided to the Cumulative Rehearsal group in the Training and Test blocks accentuated that effect, nearly doubling the frequency of rehearsals of the first three list items. This instruction did not lead participants to neglect rehearsal of items from later list positions, as they rehearsed those items as frequently as participants from the Control group.

#### Immediate Recall

**Figure 8:**
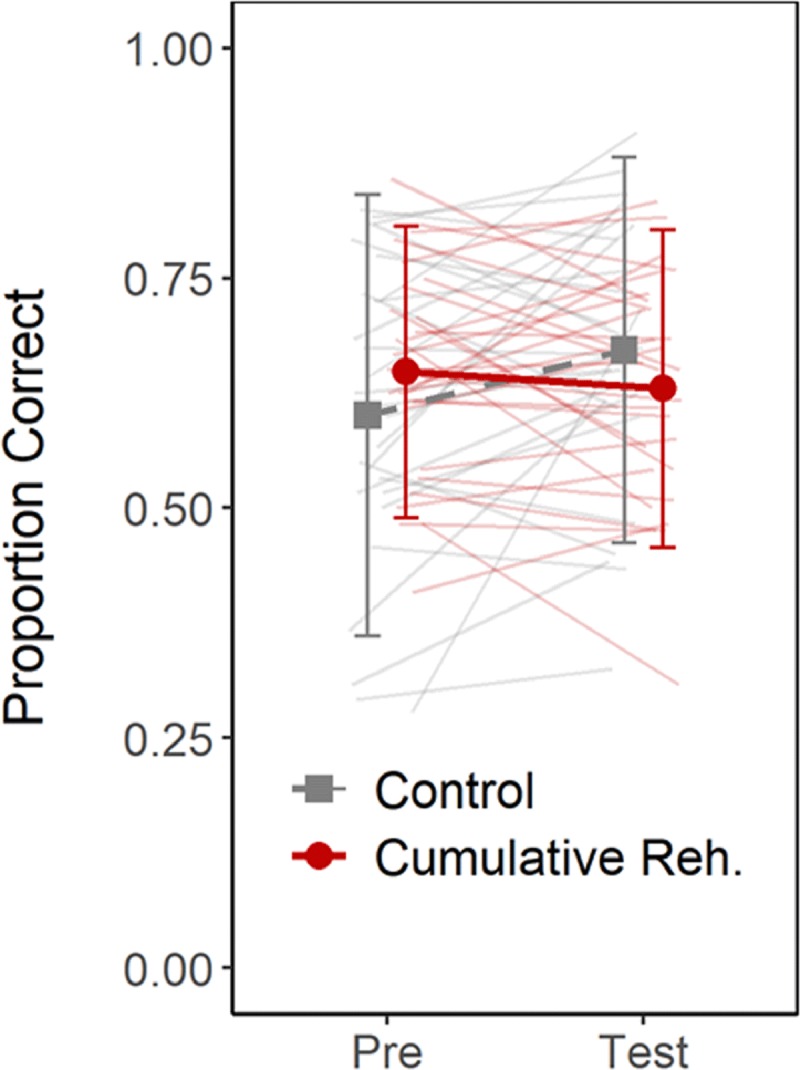
Overall proportion of correct responses in the complex span blocks (Pre-test vs. Test) in Experiment 2. Error-bars depict the standard deviation. The thin lines depict data of individual participants.

**Figure 9:**
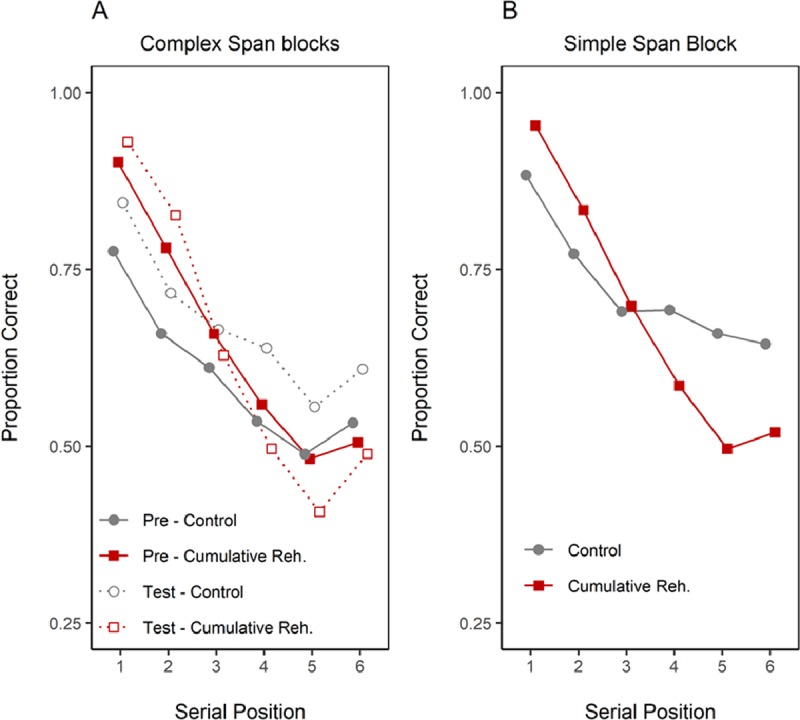
Serial position curves for the control and cumulative rehearsal groups in each block of Experiment 2. The task in Pre-test and Test blocks was complex span, and simple span in the Training block. In Training block the cumulative rehearsal group received the cumulative rehearsal instruction.

Figure 8 presents the overall recall accuracy in the Pre-test and Test blocks. Figure 9 presents the proportion of correct responses as a function of serial position. We assessed whether complex span recall changed between the Pre- and Test blocks for the Control and Cumulative Rehearsal groups. There was evidence for an interaction between block and group (BF_10_ = 5.71), but ambiguous evidence for a main effect of either block (BF_10_ = 0.75) or group (BF_10_ = 0.63). To follow-up on the interaction, we assessed the evidence for an improvement in performance between Pre-test and Test separately for each group using a one-sided t-test. For the control group, there was evidence for an improvement in complex span recall (BF_10_ = 6.7), whereas for the cumulative rehearsal group there was strong evidence against an improvement (BF_10_ = 0.10).

Figure 9 presents the serial position curves in the complex span blocks and in the simple span block. Table 6 presents the evidence for including the terms Group, Block, Serial Position, and their interactions when modeling the data of the complex span blocks. Replicating the previous analysis, there was no evidence for a main effect of group or block, whereas there was overwhelming evidence for an effect of serial position on recall. Group and block interacted (for the same reasons as detailed in the previous analysis), as did group and serial position. The latter reflects the fact that serial position curves were generally steeper for the Cumulative Rehearsal group.

**Table 6:** Bayes Factors Quantifying the Strength of Evidence for the Main Effects and Interactions of the Variables Manipulated in Experiment 2.

Term	BF_10_

Group	0.30
Block	0.57
Serial Position	1.12 × 10^48^
Group × Serial Position	19’664.2
Block × Group	5.74
Block × Serial Position	0.04
Three-way interaction	0.56

#### Rehearsal × Recall

**Figure 10:**
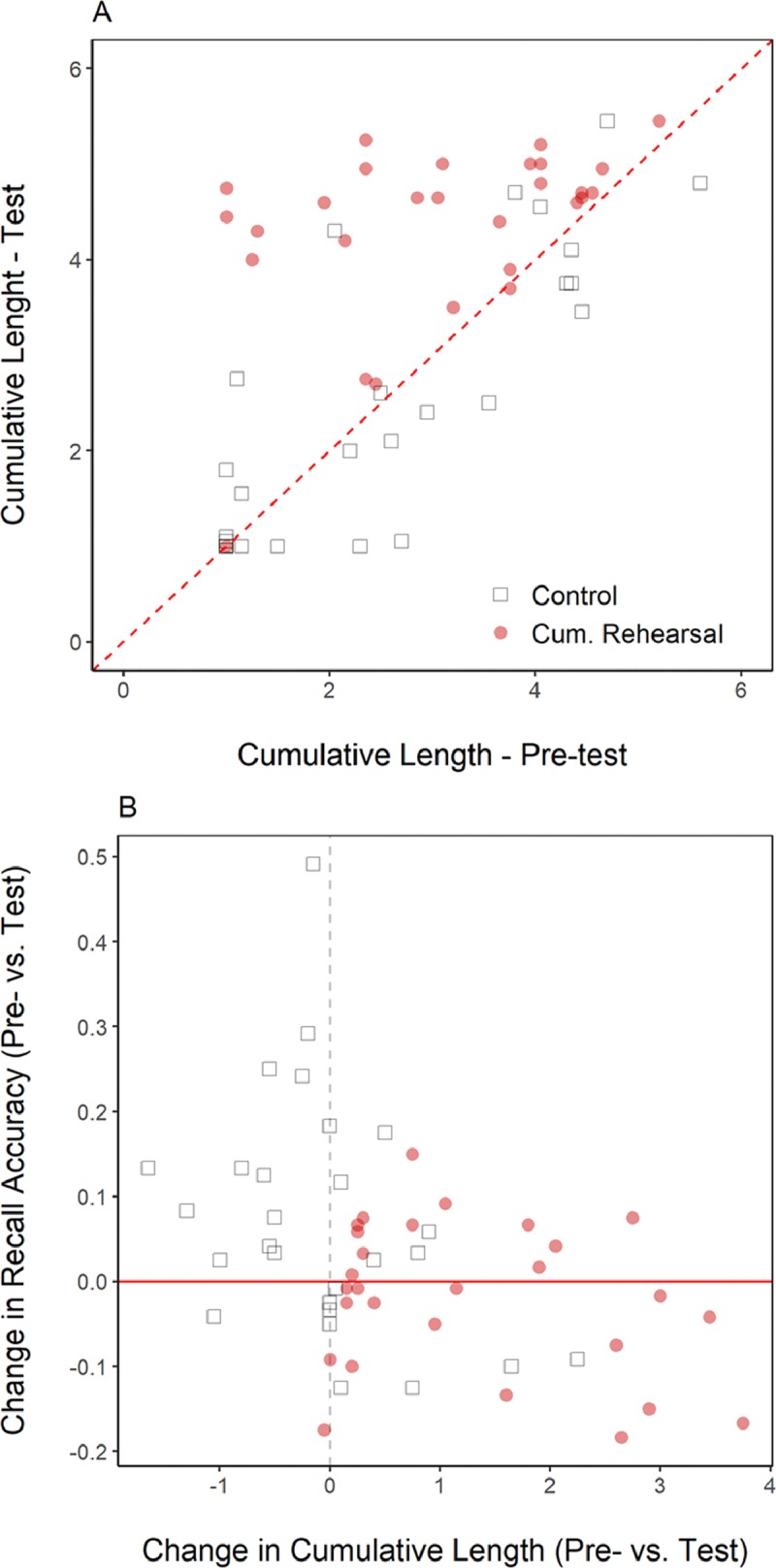
**Panel A:** average cumulative-rehearsal length in the Pre-test plotted against this measure in the Test block for each participant in Experiment 2. Values above the diagonal indicate an increase in the extent of cumulative rehearsal. **Panel B:** change in the extent of cumulative rehearsal between the Pre- and Test blocks against the change in recall accuracy between these blocks for each participant in Experiment 2.

Figure 10A presents a scatterplot relating the average length of cumulative rehearsals in the Pre-test to the average cumulative rehearsal length in the Test. Points close to the diagonal indicate little change between blocks, whereas points above the diagonal indicate increases in the extent of cumulative rehearsals between blocks. For the Control group, dots lay around the diagonal, indicating little change in cumulative rehearsal length from Pre-test to Test. Data of the Cumulative Rehearsal group, conversely, cluster above the diagonal, showing that participants in this group substantially increased the extent to which they engaged in cumulative rehearsal.

Figure 10B shows the relation between the degree of increase in cumulative rehearsal (i.e., Test vs. Pre-test) and the degree of change in recall performance between these blocks. The extent of change in cumulative rehearsal was not related to changes in recall performance for either group: For the Control group, recall increased with no change in rehearsal; for the Cumulative Rehearsal group, recall remained unchanged despite changes in the degree of cumulative rehearsal.

### Discussion

Experiment 2 assessed the causal role of cumulative rehearsals in complex span performance. Although Experiment 1 showed a positive correlation between length of cumulative rehearsal and recall accuracy in complex span, instructing and training participants to engage in this strategy in Experiment 2 had no beneficial effect for WM. This was the case despite the fact that we gradually introduced the requirements to rehearse cumulatively without the concurrent distractor task (i.e., in the Training block) to allow participants to practice and automatize this strategy. Only subsequently we asked participants to combine cumulative rehearsals with the concurrent performance of the distractor task. We reasoned this training would create favorable conditions for participants to engage in both activities without much interference, and hence to show a positive effect of rehearsal on memory.

The lack of effect on recall performance is not due to our instruction being ineffective. The instruction to perform cumulative rehearsals: (a) nearly doubled the number of words participants rehearsed; and (b) it increased the frequency and length of cumulative rehearsals. This increase was observed both in simple span (Training block) and in complex span (Test block). Although participants rehearsed much more often and managed to increase their cumulative length by about 1.3 words, rehearsal did not improve serial recall, thereby challenging the view that rehearsal and recall are causally related. If rehearsal and recall were causally related, then increasing people’s tendency to rehearse cumulatively should improve recall. Instead, rehearsal did not change how much information people could maintain in WM overall.

One may wonder whether the somewhat larger propensity of the Cumulative Rehearsal group to rehearse cumulatively already in the Pre-test compromises our conclusions. It does not, because our instruction still increased the prevalence of cumulative rehearsal substantially within the Cumulative Rehearsal group across the Pre-test and Test, whereas the Control group showed no change in rehearsal frequency and schedule. Despite changes in rehearsal, the experimental group maintained the same level of performance; conversely, the control group showed some evidence of recall improvement over blocks. This is the opposite of what would be expected under the assumption that rehearsal is beneficial to WM.

## General Discussion

People often report engaging in rehearsal during WM tasks such as simple and complex span, and many WM models assume that people rehearse because this is beneficial for WM. However, only a handful of studies have directly assessed how people rehearse in span tasks (e.g., Tan & Ward, 2008), and its causal effect on memory (Souza & Oberauer, 2018). Here we extended previous investigations using the overt rehearsal protocol in WM tasks to assess how people spontaneously rehearse in complex span tasks (Experiment 1) and the causal effect of rehearsal on recall performance in this task (Experiment 2).

We observed that participants tended to rehearse in forward cumulative order both in simple and complex span tasks. The data of Experiment 1 can be used to inform models of how people rehearse in WM tasks. Experiment 1 also showed that the extent with which participants engaged in cumulative rehearsal was correlated with their overall recall accuracy. This replicates and extends to complex span previous reports of a positive correlation between serial-recall performance and the average length of cumulative rehearsal (see also Souza & Oberauer, 2018). These correlations are compatible with the notion that cumulative rehearsal plays a beneficial role for serial recall, either by counteracting time-based decay (Baddeley, Thomson, & Buchanan, 1975; Camos et al., 2009), or by generating stronger or more robust memory representations than are produced by initial encoding (Nishiyama & Ukita, 2013; Palmer & Ornstein, 1971; Tan & Ward, 2000). The correlations, however, are equally compatible with the assumption that good memory is a prerequisite for successful cumulative rehearsal.

To determine the direction of causality between cumulative rehearsal and memory accuracy in complex span, Experiment 2 investigated the effect of instructing participants in using cumulative rehearsal. The instruction substantially increased the amount of rehearsal and the length of cumulative rehearsal sequences; nevertheless, complex span performance remained unchanged. The results of Experiment 2 disconfirm the assumption that rehearsal is beneficial. This is also in line with the results of Turley-Ames and Whitfield (2003) in which an instruction to rehearse in complex span was not beneficial when time processing the memoranda and the distractors were controlled.

Overall, our results point to the conclusion that rehearsal does not add to the strength of the memory trace in WM even if the nature of the task limits reliance on other maintenance strategies (e.g., attentional refreshing). Here we used a complex span task that limited the use of attention to reactivate memory traces, but still enabled the use of articulation. Experiment 1 showed that distractor processing does compete with the display of a cumulative rehearsal strategy. But even if participants practice and increase their use of this strategy, this added effort does not pay off for WM recall.

### Alternative explanations?

Our findings do not point to a beneficial effect of rehearsal although participants spontaneously engaged in this behavior quite often. One critique often raised against our conclusion is that people’s spontaneous rehearsal is already optimal, so that instructing them to rehearse more cannot yield a further benefit. We have two arguments against this explanation. First, if everyone spontaneously chose their optimal level of rehearsal, there should not be a positive correlation between rehearsal and recall. If rehearsal is beneficial for recall, then this correlation suggests that at least some people do not perform well *because* they were not rehearsing enough. If limited use of rehearsal explained lower recall, then increasing the extent with which participants engaged in rehearsal, as we did here, should have led to an increase in the group average performance. Our data clearly speaks against this possibility. Second, in our previous study (Souza & Oberauer, 2018) we observed that instructing people to rehearse according to the self-chosen schedule of yoked participants did not yield better performance than a matched articulatory suppression condition. Hence, even comparing a person’s spontaneous rehearsal schedule against a baseline that prevents the use of rehearsal, we found the hypothesis that rehearsal benefits WM lacking.

A related objection is that instructing people to engage in more articulatory rehearsal prevents them from engaging in other maintenance strategies such as elaboration. If this were the case, the positive correlation between length of cumulative rehearsal and recall performance would be difficult to explain. In any case, the conjecture that rehearsal competes with other, more effective maintenance strategies does not contradict our conclusion that articulatory rehearsal is not beneficial to complex-span performance – it rather offers an explanation for why it is not beneficial.

A further common objection to our findings is that we only assessed cumulative rehearsal. Critics wonder whether other rehearsal schedules could be more beneficial. Our rebuttal to this criticism is four-fold. First, we observed the spontaneous rehearsal schedules of participants, and we found no evidence that people often spontaneously engage in other patterns of rehearsal (see also Souza & Oberauer, 2018; Tan and Ward, 2008). Second, cumulative rehearsal is for theoretical reasons the schedule that should be most beneficial for performance: it is the only schedule that preserves serial order. Third, we observed correlations between serial recall and the extent of cumulative rehearsal, as did other researchers before us (Guttentag, Ornstein, & Siemens, 1987; Palmer & Ornstein, 1971; Souza & Oberauer, 2018; Tan & Ward, 2008); these correlations single out cumulative rehearsal as potentially important for WM recall. Forth, the only other strategy that was highly prevalent (i.e., Fixed rehearsals) has been found to yield costs to performance when instructed (see Souza & Oberauer, 2018).

Another common objection is that we assessed the effects of rehearsal at a memory load level that was above capacity (i.e., 6 memory items). Critics argue that rehearsal may be beneficial only when carried out within capacity (e.g. Jarrold, 2017). There are two problems with this argument. First, if memory load is within capacity, then participants can rehearse with no error, but they also can recall information perfectly, making this strategy obsolete. Second, our instruction to rehearse cumulatively increased the extent of perfect cumulative rehearsals – as indicated by the maximum cumulative rehearsal score, which indexes error-free rehearsal. Hence, if anything, our instruction pushed participants to more fully realizing their full capacity to rehearse; yet recall did not improve.

A last critique is that by pushing participants to rehearse cumulatively for longer sequences, we could have prevented the segmentation of the list into smaller groups. Grouping is known to be beneficial in simple span (Hartley, Hurlstone, & Hitch, 2016; Ryan, 1969). Some studies have observed benefits of instructing a grouping strategy (e.g., Farrell, Wise, & Lelièvre, 2011). Although grouping could be beneficial also in complex span, we don’t see evidence that our instruction changed people’s willingness to employ it. The spontaneous rehearsal data does not provide evidence that people formed and rehearsed subgroups. Participants tended to rehearse cumulatively until about the middle of the list, after which they tended to switch to a fixed rehearsal strategy. A switch to rehearsing the second half of the list would be shown as a systematic increase in rehearsals classified as “Other”. In Experiment 1, the “Other” category occurred very infrequently. In Experiment 2, the “Other” category tended to increase a bit over the last serial positions, but this tendency was similar for the Control and the Cumulative Rehearsal groups, particularly in the Test block. Given that grouping was not the target of the current research, future studies will have to examine more closely the possibility that rehearsal may help if it is combined with a grouping strategy.

### Why do people rehearse if it is not beneficial?

One reason why people may engage in rehearsal is that it increases the accessibility of item information in memory. In free recall tasks, in which serial order is not relevant, a beneficial effect of rehearsal has been observed (Laming, 2008; Rundus, 1971; Tan & Ward, 2000). Souza and Oberauer (2018) also observed a benefit of rehearsal compared to articulatory suppression when using free recall scoring of their data. Given that rehearsal is beneficial to item memory, participants may use this strategy indiscriminately, even in tasks in which this does not translate to a benefit.

Another possible explanation to the use of rehearsal is that the WM representations of verbal lists consist, at least in part, of speech plans for articulating the list. In line with ideomotor theory, a representation of an action plan in WM generates a tendency to carry it out. In a serial-recall procedure, people are instructed to withhold speaking until they are prompted to recall, so they usually suppress the overt action but nevertheless carry it out covertly (Lewandowsky & Oberauer, 2015).

## Conclusion

We found the hypothesis that articulatory rehearsal allows people to maintain more information in WM, particularly in the face of distraction as indexed by complex span performance, lacking. Our results provide a further challenge to the view that articulatory rehearsal is beneficial for WM performance.

## Data Accessibility Statements

The materials, data, and analysis scripts for the experiments reported here are available at the Open Science Framework: https://osf.io/wu3mc/. DOI: https://doi.org/10.17605/OSF.IO/WU3MC.

## Footnotes

Note that chance performance in the distractor task is 0.5; these very low values reflect mainly time-outs, or in other words, participants who ignored the distractor task in complex span.
